# Ultra-Deep DNA Methylation Analysis of X-Linked Genes: *GLA* and *AR* as Model Genes

**DOI:** 10.3390/genes11060620

**Published:** 2020-06-04

**Authors:** Giulia De Riso, Mariella Cuomo, Teodolinda Di Risi, Rosa Della Monica, Michela Buonaiuto, Davide Costabile, Antonio Pisani, Sergio Cocozza, Lorenzo Chiariotti

**Affiliations:** 1 Department of Molecular Medicine and Medical Biotechnology, Università degli Studi di Napoli ‘Federico II’, Via S. Pansini, 5, 80131 Naples, Italy; giulia.deriso@unina.it (G.D.R.); mariella.cuomo@unina.it (M.C.); buonaiutom@ceinge.unina.it (M.B.); costabile@ceinge.unina.it (D.C.); cocozza@unina.it (S.C.); 2 CEINGE Biotecnologie Avanzate, via Gaetano Salvatore 482, 80145 Naples, Italy; dirisi@ceinge.unina.it (T.D.R.); dellamonica@ceinge.unina.it (R.D.M.); 3 Department of Sanità Pubblica, Università degli Studi di Napoli ‘Federico II’, Via S. Pansini, 5, 80131 Naples, Italy; antonio.pisani@unina.it

**Keywords:** DNA methylation, X-linked genes, skewness X-inactivation, Fabry disease, epiallele analysis

## Abstract

Recessive X-linked disorders may occasionally evolve in clinical manifestations of variable severity also in female carriers. For some of such diseases, the frequency of the symptoms’ appearance during women’s life may be particularly relevant. This phenomenon has been largely attributed to the potential skewness of the X-inactivation process leading to variable phenotypes. Nonetheless, in many cases, no correlation with X-inactivation unbalance was demonstrated. However, methods for analyzing skewness have been mainly limited to Human Androgen Receptor methylation analysis (HUMARA). Recently, the X-inactivation process has been largely revisited, highlighting the heterogeneity existing among loci in the epigenetic state within inactive and, possibly, active X-chromosomes. We reasoned that gene-specific and ultra-deep DNA methylation analyses could greatly help to unravel details of the X-inactivation process and the roles of specific X genes inactivation in disease manifestations. We recently provided evidence that studying DNA methylation at specific autosomic loci at a single-molecule resolution (epiallele distribution analysis) allows one to analyze cell-to-cell methylation differences in a given cell population. We here apply the epiallele analysis at two X-linked loci to investigate whether females show allele-specific epiallelic patterns. Due to the high potential of this approach, the method allows us to obtain clearly distinct allele-specific epiallele profiles.

## 1. Introduction

More than 100 diseases are related to mutations in chromosome X genes, most of them being segregated as recessive traits [1]. In these cases, females may be asymptomatic carriers or may occasionally develop disease with highly variable clinical manifestations and severity. This phenomenon is mostly unpredictable and has been often attributed to a non-random inactivation of the female X chromosomes (skewness of X-inactivation) [2]. However, in some X-linked diseases (e.g., Fabry disease) the frequency of affected females is particularly high and only in some instances, it was explained by the skewness of X-inactivation [3]. 

Recently, the X-inactivation phenomenon has been largely revisited [4,5,6]. It was clearly demonstrated that about 15% of X-linked genes may escape from inactivation, being expressed on both active (Xa) and inactive (Xi) X chromosomes. In addition, the phenomenon of Xi escape may greatly vary not only among different individuals but also, within each individual, among tissues and even among single cells within the same tissue [7,8].

In addition, the regulatory landscape of the active X chromosome has been poorly investigated to date, also due to the relatively low resolution of the currently utilized methods of analysis. It is expected that some genes may be repressed or partially repressed on the Xa in order to be appropriately expressed in different tissues, cell types, or developmental stages. Moreover, conflicting results exist regarding the active-X two-fold gene upregulation to achieve the X-to-autosomes dosage balance in females [9]. Both the heterogeneity of the inactivation process and the active-X regulation may have a role in shaping the clinical phenotype in female carriers of X-linked diseases. 

DNA methylation is one of the best characterized epigenetic mechanisms of transcriptional regulation. Due to its key role in X-inactivation locking and maintenance, DNA methylation analysis helped to shed light on the complex epigenetic regulation of X chromosome genes [10]. 

Several studies used DNA methylation to identify genes that escape from inactivation [10,11,12] and to highlight active-X specific DNA methylation at particular loci [13].

Several methods have been also developed to assess the skewness of X inactivation on the basis of the allele differential methylation at polymorphic regions [14,15]. The current most widely used method to evaluate skewness of X-inactivation, the HUMan Androgen Receptor gene Assay (HUMARA) test, is based on the analysis of methylation status at a polymorphic region of X chromosome, specifically at the first exon of the human androgen receptor gene (*AR*) [15]. The HUMARA assay simply allows one to distinguish between maternal and paternal X chromosomes through the different numbers of CAG repeats at the high polymorphic *AR* locus. It takes advantage of the presence of several cleavage sites for methylation-sensitive restriction enzymes such as *Hpa*II in close proximity of the CAG repeats. By this way, after digestion, it is possible to amplify the *AR* locus and differentiate the methylated allele from the unmethylated one, since the HpaII enzyme cuts only when the allele is not methylated.

Because HUMARA interrogates only a few CpG sites, it may not represent the complex scenario occurring along the X chromosome, especially at X-linked genes distant from the *AR* gene. Most importantly, it is now clearly emerging that only about 50% of genes are fully inactivated while the others are variably methylated and variably expressed on the “inactive X” chromosome and possibly on the “active X” [2]. Therefore, results obtained by commonly used methods (e.g., HUMARA) give rough information about the general X-inactivation but do not give any insight into the specific, possible skewed X-inactivation at other gene loci. We reasoned that gene-specific and high-resolution DNA methylation analyses could greatly help to unravel details of the single cell X-inactivation process and the roles of specific X genes inactivation in disease manifestations. The present work describes possible novel methodologies that may help in future investigations regarding these topics.

We recently provided evidence that studying DNA methylation at specific loci at a single-molecule resolution (epiallele distribution analysis) allows one to track the spatiotemporal evolution of cell-to-cell methylation differences in a given cell population [16,17,18,19,20]. In addition to the evaluation of the average methylation, this approach considers all possible different arrangements of methylated CpG sites present in each molecule (epialleles). Each cell of a given tissue may be characterized by a specific combination of methylated CpGs at specific loci that may be related to the gene-specific functional state in that specific cell [16,17,18,19,20]. In this context, different cells with the same origin can be actually considered a mixture of epigenetically heterogeneous cells, in which each combination of methyl CpGs at a given locus represents a specific epiallele. These data are masked by evaluating the average methylation, even if evaluated at single CpG sites. Cancer cells have been also analyzed at epiallele resolution. For instance, by quantifying the entropy changes between two stages, it is possible to measure the extent of epiallele compositional change and distinguish dynamic loci that give rise to significant epiallelic shifts in leukemia and other types of cancer [21,22].

To obtain a proof of concept that this approach can be more informative and, thus, potentially give insight into X-linked genes inactivation process, we here apply the epiallele analysis at two X-linked loci in three different individual females to investigate whether females show allele-specific epiallelic patterns. Due to the high potential of this approach, the method allows us to obtain clearly distinct allele-specific epiallele profiles for each analyzed subject. Thus, we believe that epiallele analysis may be successfully applied to studies addressing important unanswered questions such as the occurrence and the variability of clinical manifestation of X-linked diseases in females, possible parental imprinting of X-linked genes, and longitudinal trajectories of X-linked epialleles and associated expression during development and aging in different individuals and different tissues.

## 2. Materials and Methods 

### 2.1. Human Whole Blood Collection 

Peripheral human whole blood from 16 women was collected from the Department of Public Health of University Federico II of Naples, Italy and stored in tubes containing EDTA at 4 °C. The participants submitted a written consent form before the blood was drawn. Human DNA was extracted from human whole blood with the Dneasy Blood & Tissue Kit (Qiagen, Hilden, Germany). The quality of DNA samples was assessed by using the instrument NanoDrop 2000 (Thermo Scientific). Sodium bisulfite conversion of genomic DNA (1 μg) was performed using commercial kit (EZ DNA Methylation Kit, Zymo Research). Converted DNA was eluted in 30 μL H_2_O. The study was approved by the local Ethical Committee (181/19) at the University of Naples Federico II.

### 2.2. Amplicon Library Preparation and Sequencing

Bisulfite-treated DNA underwent a double amplification strategy to generate an amplicon-based library. The first PCR step was performed using bisulfite-specific primers. Reactions were performed in 30 μL, total volume: 3 μL 10× reaction buffer, 0.6 μL of 10 mM dNTP mix, 0.9 μL of 5 mM forward primers (*GLA*: 5’ ATTAAGAAAGGAAGAGGGTGATTGGTT 3’; *AR*: 5’ TAAGTTTAAGGATGGAAGTGTAGTT 3’), 0.9 μL of 5 mM and reverse primers (*GLA*: 5’CTCCCAATACAACCAACCCATAATAA 3’; *AR*: 5’ CTATAAAAATTACTATTCCTCATCC 3’), 5μL bisulfite template DNA, 0.25 μL FastStart Taq, and H_2_O up to the final volume. The PCR reactions were performed with the following temperature conditions: 95 °C for 2 min; s; 38 cycles of denaturation at 95 °C for 30 s, annealing at 52 °C for 40 s, and elongation at 72 °C for 50 s; 72 °C for 6 min. After the first PCR, we performed a PCR purification using magnetic beads (Beckman-Coulter, Brea, CA). A second step of the PCR was performed to add multiplexing indices to first amplicons. The second PCR protocol was performed in 50 μL, final volume: 5 μL 10× reaction buffer, 1 μL dNTP mix (10 mM), 3 μL forward and reverse “Nextera XT” primers (Illumina, San Diego, CA), 5 μL of first PCR product, 0.4 μL KAPA Uracil plus ( BioSystem), and H_2_O up to the 50 μL final volume. The PCR reactions were performed with the following temperature conditions: 95 °C for 3 min; 8 cycles of denaturation at 98 °C for 20 s, annealing at 55 °C for 30 s, and elongation at 72 °C for 50 s; 72 °C for 5 min. AMPure purification magnetic beads (Beckman-Coulter, Brea, CA) were used to purify the PCR products were purified and Qubit® 2.0 Fluorometer instrument was used to quantify all amplicons products. An equimolar amplicon library was obtained and brought to a final concentration of 8 pM upon opportune dilution step. In order to obtain a higher variability in base calling during the sequencing, the Phix control library (Illumina) [10% (v/v)] was used. The library was subjected to paired-end sequencing. V2 reagents kit (Illumina) was used for sequencing reactions. 

### 2.3. HUMARA Assay

HUMARA assay was performed according to a previously reported protocol [13]. Accordingly, 2 μg of genomic DNA of each sample were digested with *Hpa*II for 12 hours at 37 °C. To inactivate the enzyme activity, we incubated the samples at 95 °C for 10 minutes. PCR amplification of the *AR* gene was performed using as a template both the digested DNA and undigested DNA. The amplification protocol was: 3 μL 10x reaction buffer; 6 μL forward and reverse primers 5 μM (FW: 5’ GGGAAGGGTCTACCCTCGGCCGCC 3’; RV: 5’ CGATGGGCTTGGGGAGAACCATCC 3’); 1 μL dNTPs 10mM; 0,3 μL hotStart Taq (Biorad); H_2_O up to the 30 μL final volume. The PCR reactions were performed with the following temperature conditions: 95 °C for 15 min; 27 cycles of denaturation at 95 °C for 30 s, annealing at 60 °C for 40 s and elongation at 72 °C for 1 min; 72 °C for 10 min. Agarose gel (5%) electrophoresis was used to analyze the amplification products. Electrophoretic run for 16 hours at 50 V. ImageJ software was used to quantify the PCR products. The skewness of X-inactivation was quantified using the following formula [15]:

$$\frac{\frac{d1}{u1}}{\frac{d1}{u1}+\frac{d2}{u2}}$$

where d1 and u1 indicate the band densities after background subtraction of the longer allele (34 repeats) from the digested and undigested sample respectively, while d2 e u2 represent the band densities of the shorter allele (26 repeats).

### 2.4. Sequence Handling

We performed a quality check of the paired-end reads obtained from the Illumina Miseq sequencer using the fastQC tool. The average length of the reads was 247 bps and the average quality score x read was 33. A minimum of 11655 reads were obtained for the sequenced samples. Detailed per sample information of read length, quality score x sequence, and the total number of reads are reported in Table S1.

The forward and reverse reads from each sample were merged using the PEAR tool with the following parameters: (1) a minimum overlap of 40 bases, (2) minimum read Phred score [23] of 33. The fastq files containing the assembled reads were converted to the FASTA format with the PRINSEQ tool. Sequences were submitted to ad-hoc R scripts that: (1) searched for the *AR* and *GLA* genetic polymorphisms to find heterozygous females at both loci in the screened population. Briefly, we searched for a consensus sequence in the FASTA files consisting of the sequence of the searched variant plus the 5’ and 3’ unique flanking regions. We considered a subject homozygous for the *AR* locus if the reads in the relative FASTA file matched two different consensus sequences differing for the number of CAG repeats. For the *GLA* locus, we considered a subject homozygous if the FASTA reads matched the consensus of both the wild-type and the alternative allele of the searched SNP; (2) sorted the bisulfite reads in heterozygous individuals according to their sequence. In this way, six FASTA files were generated for each locus, containing the sequences bearing a specific polymorphism. The resulting FASTA files were then independently analyzed. We also deposited the raw sequences in the European Nucleotide Archive database with the accession number: PRJEB37857

### 2.5. High Coverage-Amplicon Bisulfite Sequencing

We analyzed the methylation profiles of the reads from each individual FASTA file using the AmpliMethProfiler Tool [18] freely available at https://sourceforge.net/projects/amplimethprofiler. As a first step, the tool aligned each read to the amplicon reference sequence using the Blastn tool. Reads that only partially matched the reference sequence were discarded. Then, the pipeline checked the efficiency of the bisulfite conversion of each read, looking at the base aligned with the non-CpG cytosines in the reference sequence. Only the reads with more than 98% of converted non-CpG cytosines were retained. Finally, the status of the CpG cytosines was inspected. If a C was found in the correspondent position of the aligned read, the site was considered methylated; if, instead, a T was found, the site was considered unmethylated; all the reads bearing a gap or other bases (A or G) in a CpG position were discarded. At the end, we obtained a CpG methylation profile matrix, with rows representing the retained reads in the FASTA file and columns representing the CpG sites. Each row, indeed, corresponded to the methylation profile of a single DNA molecule, which we refer to as epiallele.

To analyze an equal number of methylation profiles (epialleles) for the locus-paired alleles, we performed a rarefaction procedure, randomly selecting from the allele showing the highest-coverage the same number of profiles of the one with the lowest-coverage. Details of the subject and locus descriptions of the number of analyzed epialleles after this rarefaction procedure can be found in Table S1.

The following parameters were calculated for the analyzed regions on the basis of the information from the methylation matrices:

1) Skewness of inactivation:
  $$\frac{\mathrm{Ma}1}{\left(\mathrm{Ma}1+\mathrm{Ma}2\right)\;}$$
  where Ma1 indicates the total number of methylated CpGs on the *AR* longer allele and Ma2 the total number of methylated CpGs on the shorter one.
2) Allele average methylation: for each analyzed allele, the average methylation was calculated as
  $$\frac{\mathrm{Ma}}{\mathrm{Na}}$$
  where Ma indicates the total number of allele methylated CpGs and Na indicates the total number of assayed CpGs. Locus average methylation was calculated averaging the methylation level found on each CpG on each allele, respectively.
3) CpG average methylation: For each allele independently, the average methylation of a CpG site was calculated as
  $$\frac{M\left(\mathrm{CpG},\;a\right)}{\mathrm{Ea}}$$
  where M(CpG,a) indicate the number of molecules in which the CpG is methylated, while Ea is the total number of analyzed epialleles.

### 2.6. Statistical Analysis

All statistical analyses were performed using R software (version 3.6.0 [24]) with a α value set for *p-*value < 0.01.

## 3. Results and Discussion

**Figure 1:**
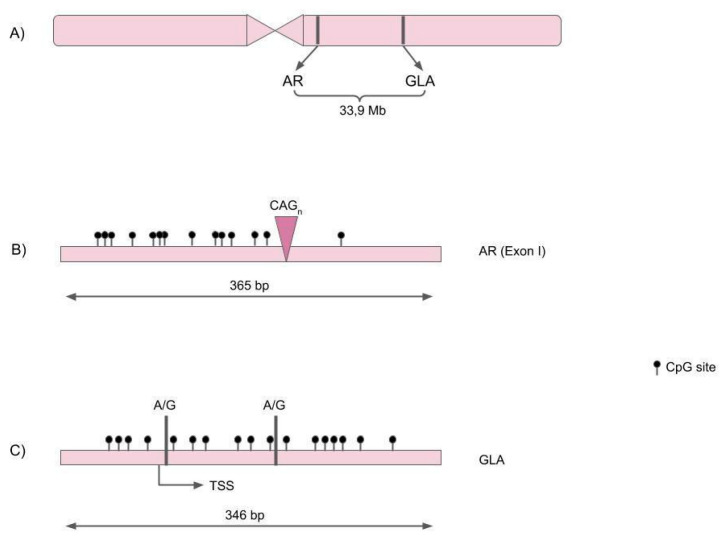
(**A**) Androgen Receptor (*AR*) and α-galactosidase (*GLA*) gene position on the X chromosome. (**B**) Structure of the *AR* amplicon (365 bps; hg19 genomic coordinates: 66764978-66765342, strand+). The position of the trinucleotide repeat (CAG) is indicated. (**C**) Structure of the *GLA* amplicon (346 bps; hg19 genomic coordinates: 100662748-100663093, strand-). The position of the single nucleotide polymorphisms (A/G) and of the transcription start site (TSS) is indicated.

In order to compare HUMARA and High Coverage-Amplicon Bisulfite Sequencing (*HC-ABS)* approaches, we selected two representative gene loci on the X-chromosome. *GLA* and *AR* were chosen as the models of the X chromosome genes on the basis of the following criteria: (1) the presence of a genetic variant that we could use as an allelic marker in heterozygous females; (2) a minimum distance between the loci, so that they could be considered as independent elements. The first region that we selected is on the long arm of the X chromosome (q1.2, hg19: 66764978-66765342, strand+), in the first exon of the androgen receptor (*AR*) gene. It includes 14 CpG sites flanking the *AR* trinucleotide repeat (CAG) (Figure 1B). This polymorphism has been widely used for X chromosome allelic discrimination, for example in the HUMARA test, thus enabling us to compare the results from *HC-ABS* with this standard technique.

The second region is also located in the X chromosome long arm (q 2.22.1, hg19: 100662748-100663093, strand-) and lies about 33 Mb distant from the *AR* locus (Figure 1A). This region surrounds the alpha galactosidase gene transcription start site, and includes 17 CpGs flanking two potentially informative single nucleotide polymorphisms: rs3027583 A/G (frequency of heterozygosity: 0.03 in dbSNP 151) and rs3027585 A/G (frequency of heterozygosity: 0.06 in dbSNP 151) (Figure 1C).

**Figure 2:**
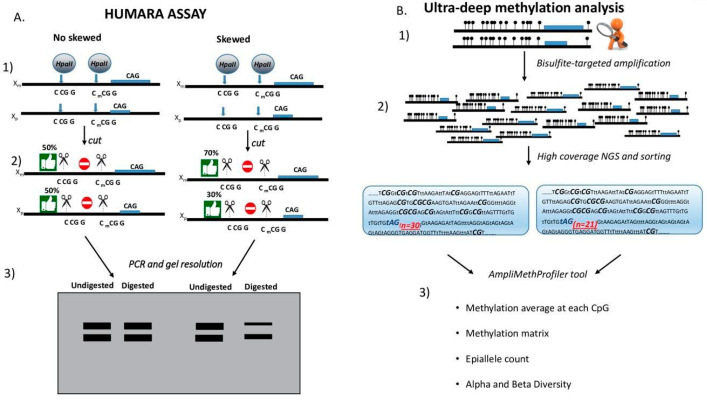
Comparison between Human Androgen Receptor methylation analysis (HUMARA) and ultra-deep methylation sequencing. (**A**) HUMARA strategy to verify skewness on *AR* locus. On the left, an example of *AR* locus with no skewness. On the right, an example of the *AR* locus with skewness. (1) *AR* locus with two sites for HpaII restriction enzyme located upstream of the CAG repeats region. The two X alleles are indicated as Xm and Xp. (2) HpaII enzyme cuts CCGG nucleotides only when the dimer CG is not methylated. On the left, the enzyme HpaII can cut on 50% of molecules, both on Xp and on Xm. On the right, the cutting capacity of the enzyme is on 70% of alleles belonging to Xm and on 30% of the alleles belonging to Xp. (3) Agarose gel after PCR of the two putative samples. Undigested and digested samples are shown. Thanks to the differences in CAG repeats on the two alleles, it is possible to distinguish on the agarose gel the Xm and Xp. On the left, where there is no skewness, the two bands show the same intensity, since the cut occurs on the same percentage of molecules both in Xp and in Xm. On the right, the shorter allele shows a lower intensity than the longer one, since the cut occurs on 30% of Xp and on 70% of Xm. (**B**) Ultra-deep methylation strategy on the *AR* locus. (1) The *AR* amplicon is analyzed by *HC-ABS*. All the analyzed CpG sites are shown as black circles. Different CAG repeats are indicated for Xm and Xp. (2) Amplicon library is prepared and sequenced by Next Generation Sequencing (NGS). The number of repeated CAGs are indicated in red. CpG dimers are indicated in bold. After sequencing, the two alleles are sorted according to the different number of repeated CAGs. (3) Separated alleles are analyzed with the AmpliMethProfiler Tool [18]. After bioinformatic analysis, the methylation average at each CpG, methylation matrix, epiallele count and alpha and beta diversity are obtained for each separated allele.

To compare the DNA methylation pattern between the two X alleles, we screened a group of 16 females and identified three heterozygous at both loci individuals belonging to the same family. (data not shown). In particular, these individuals shared one *AR* allele with 26 CAG repeats while this differed for the number of repeats of the second allele. For the *GLA* region, all the three individuals were heterozygous for the rs3027585 SNP. Knowing the respective father’s genotype (*GLA*:A, *AR*:26 repeats) enabled us to reconstruct the phase of the X-chromosome haplotype (*AR*:26,*GLA*:A on the paternal X and *AR*:34/23/22,*GLA*:G on the maternal X). A schematic comparison of the HUMARA and *HC-ABS* approaches is depicted in Figure 2.

### 3.1. Deep-Bisulfite Sequencing Enables a Consistent Estimation of the Skewness 

**Figure 3:**
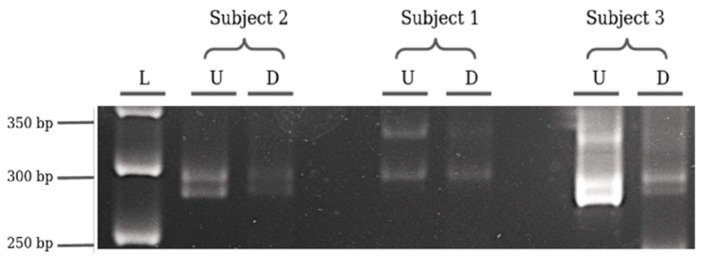
HUMARA test results. L: 50 bp Biolabs molecular marker; U: undigested subjects, D: digested subjects. Subject 2 amplicons length: allele 1 (26 repeats) 291 bp, allele 2 (22 repeats) 282 bp; subject 1 amplicons length: allele 1 (26 repeats) 291 bp, allele 2 (34 repeats) 324 bp; subject 3 amplicons length: allele 1 (26 repeats) 291 bp, allele 2 : (23 repeats) 286bp.

Firstly, the HUMARA test on the *AR* locus was used as a reference to assess the presence of a skewed X inactivation in the three selected subjects. The PCR products were quantified using ImageJ software. After the background subtraction, we calculated the methylated fraction of each allele as the ratio between the digested and the undigested band densities. Assuming that the methylated fraction of an allele resembles the inactivated quote of such allele, we estimated the skewness of the X-inactivation as the ratio between the methylated fraction of the longer allele and the methylated fraction of the locus (calculated as the sum of the methylated fraction of both alleles, see Materials and Methods for detailed formulae). Thus, values shown in Table 1 correspond to the percentage of methylation of the longer allele for each subject (e.g., subject 1: longer allele value: 0.22 = 22%; as a consequence, shorter allele value: 1 − 0.22 = 0.78 = 78%) (Figure 3). 

We then performed the methylation analysis of the same region at the *AR* locus and at the *GLA* promoter by *HC-ABS*. For each locus, after the bisulfite sequencing, we sorted the obtained reads according to the presence of either *AR* and *GLA* polymorphisms. Two FASTA files were generated for each locus, containing sequences bearing a specific polymorphism. The four resulting FASTA files were then independently analyzed. We determined the asset of methylated and unmethylated CpG sites of each read (named epialleles) using the AmpliMethProfiler Tool [18]. We performed a random sampling step (rarefaction procedure, see Materials and Methods) to analyze an equal number of epialleles for either the *AR* and *GLA* alleles. The per-subject number of analyzed epialleles is reported in Table S1. For the *AR* locus, we quantified the skewness of the X-inactivation at the targeted regions by averaging the number of methylated cytosines on the longer allele (allele 34 for subject 1, allele 26 for subjects 2 and 3) for the total number of methylated cytosines on both alleles (see Materials and Methods). 

Comparing the results from *HC-ABS* with the HUMARA ones at the *AR* region, we found a substantial concordance in the skewness direction. For subject 1, the *HC-ABS* method estimated a lower value of *AR* allelic imbalance compared to the HUMARA (25/75 vs 35/65). We speculated that, in addition to differences in the sensitiveness of the two techniques, the different number of CpGs on which the two methods rely could ultimately result in the observed variations. Indeed, only two dinucleotides (CpGs 66765098 and 66765138 in the *AR* amplicon) were evaluated in the HUMARA test, due to the *Hpa*II restriction site constrain. On the contrary, the *HC-ABS* method performed a single molecule analysis of the methylation status of all 14 the CpG sites in the analyzed region.

Using the same method, we quantified the skewness of the X-inactivation at the *GLA* locus. To compare the results with those at the *AR* locus, we calculated the skewness towards the *GLA* allele in the phase with the *AR* longest allele (allele G for subject 1, allele A for subjects 2 and 3). We observed a substantial concordance in the direction and entity of the methylation imbalance between the two loci. In particular, we observed a preferential methylation of the paternal allele at both loci. These results suggested that the analysis of X chromosome loci through *HC-ABS* can identify an imbalance of X-inactivation. Moreover, this method is not limited to the *AR* gene but can be directly performed on other loci of interest. 

**Figure 4:**
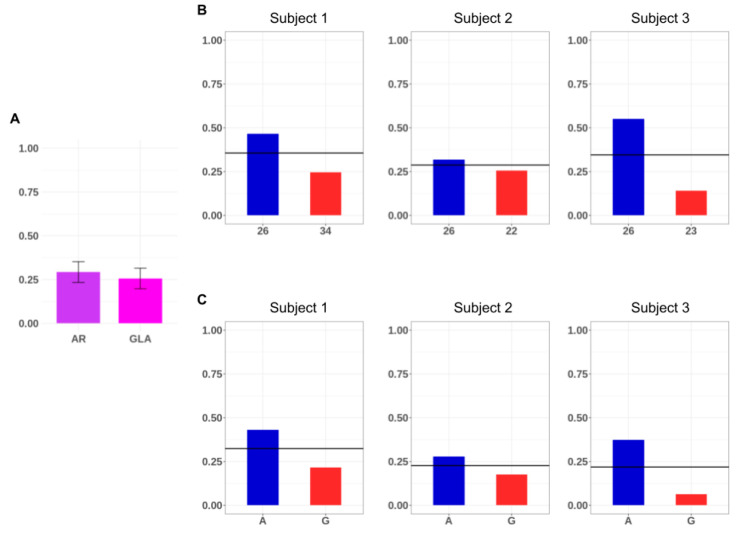
Locus and allele-specific average DNA methylation of the analyzed regions. (**A**) Locus average DNA methylation of the *AR* and *GLA* analyzed regions in the three selected subjects. The bars indicate the standard deviation between the subjects. (**B**) Allele-specific DNA methylation of the androgen receptor (*AR*) for the three subjects. The paternal 26 allele is colored in blue; the maternal alleles (34 for subject 1, 22 for subject 2 and 23 for subject 3) are colored in red. (**C**) Allele-specific DNA methylation of the alpha galactosidase (*GLA*) amplicon for the three subjects. The paternal shared A allele is colored in blue, while the maternal G allele in blue. In (**B**) and (**C**) the *AR* and *GLA* locus average methylation value is indicated (horizontal black line).

For each locus (combining both alleles), we then calculated the average DNA methylation as the number of methylated cytosines over the total number of assayed cytosines (methylated and non-methylated). We calculated the DNA methylation values obtained from the DNA methylation average of the three analyzed subjects without separating the two alleles (Figure 4A). Considering each allele individually, we also calculated the allelic average DNA methylation for each subject (Figure 4B,C).

As shown in Figure 4A, the *GLA* promoter exhibited a slightly lower total methylation than the *AR* (*AR* average methylation: 0.33, standard error = 0.02, *GLA*: 0.25, standard error = 0.03) Regardless of these little methylation differences between loci, skewness showed very similar values. This information cannot be retrieved from the HUMARA results and could be of help when analyzing the impact of the skewness on the phenotype.

### 3.2. HC-ABS Method Identified DNA Methylation Differences Between the Alleles at a Single CpG Level.

**Figure 5:**
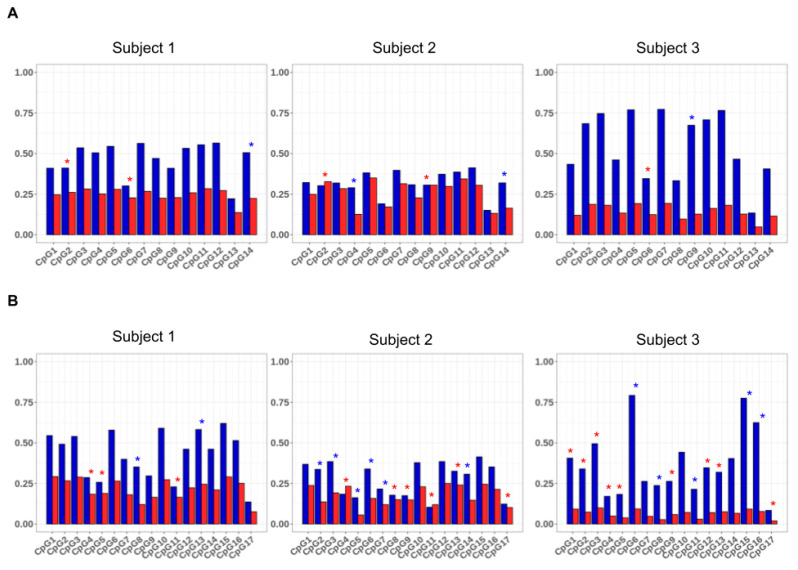
Allelic average methylation of the *AR* (**A**) and *GLA* (**B**) CpG dinucleotides. For a specific CpG, the allele average methylation was calculated as the number of times the CpG was methylated on the allele compared to the total number of epialleles. CpGs with a significant difference in the average methylation between the two alleles are indicated (Χ^2^ with Bonferroni–Holm post-hoc test *p*-values <0.01) with an asterisk. The color of the asterisks indicates the allele with a positive residual from the post-hoc test (more methylated). Blue asterisks: CpGs with post-hoc test *p*-value <0.01 and with a positive residual for the paternal allele. Red asterisks: CpGs with post-hoc test *p*-value <0.01 and with a positive residual for the maternal allele.

The use of the *HC-ABS* method enabled us to investigate whether each allele, independently of the inactivation imbalance, tended to be methylated at the same CpG sites. For this aim, we calculated for each allele individually the average methylation of the individual CpGs as the number of methylation events observed for the CpG on the number of epialleles. The resulting values for the three subjects are shown in Figure 5. In all the subjects, the analyzed CpGs were methylated to different extent on the alleles of either *AR* and *GLA*, some dinucleotides being more frequently modified than others. Furthermore, the average values of methylation differed between the two alleles (*p*-value <0.01, Table S1). In particular, several CpGs in the *AR* gene and *GLA* genes exhibited a statistically significant allelic preferential methylation in the analyzed subjects (Bonferroni–Holm post-hoc test <0.01) Interestingly, this allelic preferential methylation was also observed in subject 2, in which the allelic average methylation was quite balanced. 

**Figure 6:**
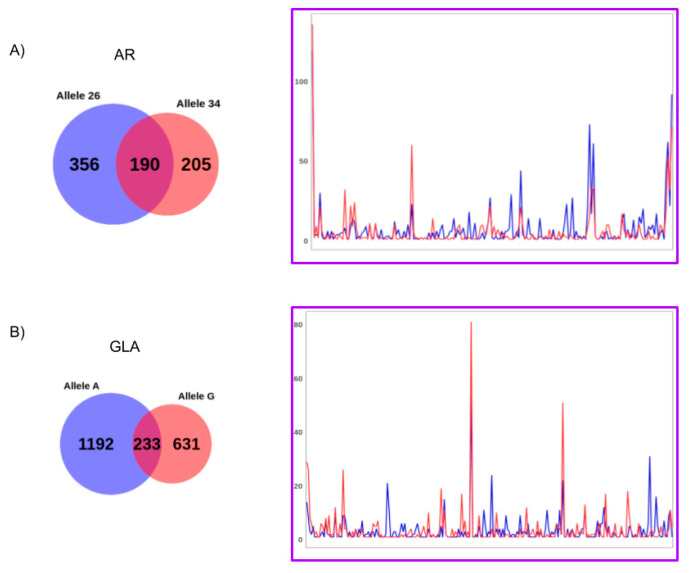
Results of the epialleles analysis for the *AR* (**A**) and *GLA* (**B**) allelic sequences from subject 1. The Venn diagrams summarize the number of allele-specific (red and blue circles) and shared (purple circle) epialleles. For the shared epialleles (excluding the fully unmethylated epiallele), the allelic counts are shown (red and blue lines). The epialleles are ordered on the *x*-axis based on the increased number of methylated sites.

Considering the observed allelic heterogeneity in the CpG methylation status, analyzing the total number of CpGs in a region can result in a more robust estimation of the skewness then considering only a few CpGs. Moreover, further information could be obtained by the analysis of the difference of the epialleles’ distribution between the two alleles. Indeed, upon high coverage bisulfite sequencing, it is possible to investigate either the asset of methylated and unmethylated CpG sites present in each amplicon-derived sequence with high precision. As an example, in a mixed population of cells, the analyzed region of *AR* gene, which includes 14 CpG sites, may give origin to 2^14^ (16,384) possible combinations, while the analyzed region of *GLA* gene may generate 2^17^ (131,072). These different combinations are here referred to as “epialleles”, where the amount of different exhibited epialleles gives a measure of “epipolymorphism”. We tentatively performed the epiallele distribution analysis of *GLA* and *AR* genes for each individual subject, considering the polymorphic paternal and maternal alleles as separate objects. The results for subject 1 are shown in Figure 6 and described here below. Those for subjects 2 and 3 are reported in Figures S1 and S2. As the coverage obtained in this study was very high but still limited compared to the total number of possible epialleles, we expected that all the epialleles eventually represented at very low frequency will be missing from the count and therefore filtered out. 

A total of 751/16,384 different epialleles for the *AR* locus and 2056/131,072 different epialleles for the *GLA* locus were found in subject 1. Most of these epialleles were allele-specific, while only 190 epialleles for the *AR* and 233 for *GLA* gene were identified as shared by both the parental alleles, respectively. These shared epialleles likely represents the “core” group of epialleles that are represented independently on the average methylation levels (unbalanced in the case study), parental origin, or genetic polymorphisms present on each analyzed allele. Interestingly, when comparing the counts, also this group of common epialleles exhibited different profiles between the alleles (Figure 5; Table S2) highlighting the potential value of the approach here utilized to distinguish the epiallele distribution profiles of X chromosome associated alleles. 

In this work, we have not discriminated Xa and Xi methylation profiles of the model genes by performing distinct analyses. Rather, we have treated *AR* and *GLA* genes as “autosomal” genes and performed the methylation analyses on individual polymorphic alleles on the basis of the assumption that a cell-to-cell variability may exist in the gene-specific methylation pattern among individual Xa and Xi [7]. The analysis gave us a landscape of the variegate epiallelic compositions of each allele for each selected locus, thus providing evidence that this approach has high potentiality for the determination of the degree of epipolymorphism and for evaluating the effective percentage of active alleles for each locus. 

We and others have recently shown that the epiallele distribution profiles of autosomal genes is generated in a very well-orchestrated manner and follow precise spatiotemporal-dependent trajectories [15,16,17,19,25]. These epiallele profiles are likely influenced also by other genetic factors, such as local polymorphisms, or environmental factors involving potential individual- and allele-specific trajectories. The limitation of this study is the low number of analyzed subjects that did not allow us to perform effective statistical analyses. However, the here reported data provide a proof of concept that an ultradeep methylation analysis of X-linked genes may represent a new tool to investigate the phenomena underlying the interindividual phenotypic variations and, perhaps, to predict the tendency to develop clinical symptoms of recessive X-linked disease in females. This would be of particular importance, since for some X-linked diseases a therapy exists (e.g., Fabry disease), but it is administered in female carriers only at the onset of symptoms. Tracking multiple epiallele distribution profiles on several symptomatic or asymptomatic female carriers of specific diseases, coupled to ad hoc bioinformatic analyses aimed at comparing the groups’ profiles [16,17,18,20], will be necessary in the near future for such prediction attempts.

## Figures and Tables

**Table 1:** Quantification of the skewed X-inactivation at the *AR* and *GLA* target regions. Quantifications of the skewness at the *AR* locus calculated using the HUMARA and the *HC-ABS* methods are reported in the first column and in the second column, respectively. Quantification of the skewness at the *GLA* locus calculated with the *HC-ABS* method is reported in the third column.

	*AR* HUMARA	*AR HC-ABS*	*GLA HC-ABS*
Subject 1	0.22	0.34	0.33
Subject 2	0.53	0.55	0.61
Subject 3	0.73	0.79	0.85

## Supplementary Materials

The following are available online at https://www.mdpi.com/2073-4425/11/6/620/s1, Figure S1: Results of the epialleles analysis for the AR (A) and GLA (B) allelic sequences from subject 2. Figure S2: Results of the epialleles analysis for the AR (A) and GLA (B) allelic sequences from subject 3. Table S1: Sequence statistics and experiments raw data. Table S2: Common epiallele counts subjects 2 and 3.

## References

[1] Germain D.P. (2006). Fabry Disease: Perspectives from 5 Years of FOS.

[2] Minks J., Robinson W.P., Brown C.J. (2008). A skewed view of X chromosome inactivation. J. Clin. Investig..

[3] Wang R.Y., Lelis A., Mirocha J., Wilcox W.R. (2007). Heterozygous Fabry women are not just carriers, but have a significant burden of disease and impaired quality of life. Genet. Med..

[4] Balaton B.P., Dixon-McDougall T., Peeters S.B., Brown C.J. (2018). The eXceptional nature of the X chromosome. Hum. Mol. Genet..

[5] Berletch J.B., Yang F., Disteche C.M. (2010). Escape from X inactivation in mice and humans. Genome Boil..

[6] Vacca M., Della Ragione F., Scalabrì F., D’Esposito M. (2016). X inactivation and reactivation in X-linked diseases. Semin. Cell Dev. Boil..

[7] Keniry A., Blewitt M.E. (2018). Studying X chromosome inactivation in the single-cell genomic era. Biochem. Soc. Trans..

[8] Garieri M., Stamoulis G., Blanc X., Falconnet E., Ribaux P., Borel C.A., Santoni F., Antonarakis S.E. (2018). Extensive cellular heterogeneity of X inactivation revealed by single-cell allele-specific expression in human fibroblasts. Proc. Natl. Acad. Sci. USA.

[9] Veitia R.A., Veyrunes F., Bottani S., Birchler J.A. (2015). X chromosome inactivation and active X upregulation in therian mammals: Facts, questions, and hypotheses. J. Mol. Cell Boil..

[10] Sharp A.J., Stathaki E., Migliavacca E., Brahmachary M., Montgomery S.B., Dupré Y., Antonarakis S.E. (2011). DNA methylation profiles of human active and inactive X chromosomes. Genome Res..

[11] Cotton A.M., Price M., Jones M., Balaton B.P., Kobor M.S., Brown C.J. (2014). Landscape of DNA methylation on the X chromosome reflects CpG density, functional chromatin state and X-chromosome inactivation. Hum. Mol. Genet..

[12] Moen E., Litwin E., Arnovitz S., Zhang X., Zhang W., Dolan M.E.A., Godley L. (2015). Characterization of CpG sites that escape methylation on the inactive human X-chromosome. Epigenetics.

[13] Joo J.E., Novakovic B., Cruickshank M., Doyle L.W., Craig J.M., Saffery R. (2014). Human active X-specific DNA methylation events showing stability across time and tissues. Eur. J. Hum. Genet..

[14] Hatakeyama C., Anderson C., Beever C., Peñaherrera M., Brown C.J., Robinson W.P. (2004). The dynamics of X-inactivation skewing as women age. Clin. Genet..

[15] Allen R.C., Zoghbi H.Y., Moseley A.B., Rosenblatt H.M., Belmont J.W. (1992). Methylation of HpaII and HhaI sites near the polymorphic CAG repeat in the human androgen-receptor gene correlates with X chromosome inactivation. Am. J. Hum. Genet..

[16] Cuomo M., Keller S., Punzo D., Nuzzo T., Affinito O., Coretti L., Carella M., De Rosa V., Florio E., Boscia F. (2019). Selective demethylation of two CpG sites causes postnatal activation of the Dao gene and consequent removal of d-serine within the mouse cerebellum. Clin. Epigenet..

[17] Keller S., Punzo D., Cuomo M., Affinito O., Coretti L., Sacchi S., Florio E., Lembo F., Carella M., Copetti M. (2018). DNA methylation landscape of the genes regulating D-serine and D-aspartate metabolism in post-mortem brain from controls and subjects with schizophrenia. Sci. Rep..

[18] Scala G., Affinito O., Palumbo D., Florio E., Monticelli A., Miele G., Chiariotti L., Cocozza S. (2016). ampliMethProfiler: A pipeline for the analysis of CpG methylation profiles of targeted deep bisulfite sequenced amplicons. BMC Bioinform..

[19] Affinito O., Scala G., Palumbo D., Florio E., Monticelli A., Miele G., Avvedimento V.E., Usiello A., Chiariotti L., Cocozza S. (2016). Modeling DNA methylation by analyzing the individual configurations of single molecules. Epigenetics.

[20] Florio E., Keller S., Coretti L., Affinito O., Scala G., Errico F., Fico A., Boscia F., Sisalli M.J., Reccia M.G. (2016). Tracking the evolution of epialleles during neural differentiation and brain development: D-Aspartate oxidase as a model gene. Epigenetics.

[21] Li S., Garrett-Bakelman F., Perl A.E., Luger S.M., Zhang C., To B.L., Lewis I.D., Brown A.L., D’Andrea R.J., Ross M.E. (2014). Dynamic evolution of clonal epialleles revealed by methclone. Genome Biol..

[22] Jenkinson G., Pujadas E., Goutsias J., Feinberg A.P. (2017). Potential energy landscapes identify the information-theoretic nature of the epigenome. Nat. Genet..

[23] Ewing B., Hillier L., Wendl M.C., Green P. (1998). Base-calling of automated sequencer traces using Phred. I. Accuracy Assesment. Genome Res..

[24] R Core Team (2017). A Language and environment statistical computing. R Foundation For Statistical Computing, Vien, Austria. https://www.R-project.org.

[25] Onuchic V., Lurie E., Carrero I., Pawliczek P., Patel R.Y., Rozowsky J., Galeev T., Huang Z., Altshuler R., Zhang Z. (2018). Allele-specific epigenome maps reveal sequence-dependent stochastic switching at regulatory loci. Science.

